# Recurrence of pulmonary tuberculosis in India: Findings from the 2019–2021 nationwide community-based TB prevalence survey

**DOI:** 10.1371/journal.pone.0294254

**Published:** 2023-12-21

**Authors:** Prathiksha Giridharan, Sriram Selvaraju, Raghuram Rao, Kiran Rade, Kannan Thiruvengadam, Smita Asthana, Rakesh Balachandar, Sampada Dipak Bangar, Avi Kumar Bansal, Jyothi Bhat, Debjit Chakraborty, Vishal Chopra, Dasarathi Das, Shanta Dutta, Kangjam Rekha Devi, Sunil Kumar, Avula Laxmaiah, Major Madhukar, Amarendra Mahapatra, Suman Sundar Mohanty, Chethana Rangaraju, Jyotirmayee Turuk, Kamran Zaman, Rajendran Krishnan, Sivakumar Shanmugam, Nishant Kumar, Rajendra Panduranga Joshi, Somashekar Narasimhaiah, Padmapriyadarsini Chandrasekaran, Raman R. Gangakhedkar, Balram Bhargava

**Affiliations:** 1 ICMR- National Institute for Research in Tuberculosis, Chetpet, Chennai, India; 2 Central TB Division, Ministry of Health and Family Welfare, New Delhi, India; 3 National Professional Officer, WHO Country Office, New Delhi, India; 4 ICMR- National Institute for Cancer Prevention and Research, Noida, Uttar Pradesh, India; 5 ICMR- National Institute for Occupational Health, Ahmedabad, Gujarat, India; 6 ICMR- National AIDS Research Institute, Pune, Maharashtra, India; 7 ICMR- National JALMA Institute of Leprosy and other Mycobacterial diseases, Agra, Uttar Pradesh, India; 8 ICMR- National Institute for research in Tribal Health, Jabalpur, Madhya Pradesh, India; 9 ICMR- National Institute of Cholera and Enteric Diseases, Kolkata, India; 10 State TB Training and Demonstration Centre (STDC), TB Hospital, Lahori, Punjab, India; 11 ICMR- Regional Medical Research Centre, Bhubaneshwar, Odisha, India; 12 ICMR- Regional Medical Research Centre, Dibrugarh, Assam, India; 13 State TB Cell, Trivandrum, Kerala, India; 14 ICMR- National Institute for Research in Nutrition, Hyderabad, Telangana, India; 15 ICMR- Rajendra Memorial Research Institute of Medical Sciences Agamkuan, Patna, India; 16 ICMR- ICMR-National Institute for Implementation Research on Non-Communicable Diseases, Jodhpur, India; 17 National Tuberculosis Institute, Bengaluru, Karnataka, India; 18 ICMR- Regional Medical Research Centre, Gorakhpur; 19 Indian Council of Medical Research, New Delhi, India; The Foundation for Medical Research, INDIA

## Abstract

Recurrent Tuberculosis patients contribute to a significant proportion of TB burden in India. A nationwide survey was conducted during 2019–2021 across India among adults to estimate the prevalence of TB. A total of 322480 individuals were screened and 1402 were having TB. Of this, 381 (27.1%) had recurrent TB. The crude prevalence (95% CI) of recurrent TB was 118 (107–131) per 100,000 population. The median duration between episodes of TB was 24 months. The proportion of drug resistant TB was 11.3% and 3.6% in the recurrent group and new TB patients respectively. Higher prevalence of recurrent TB was observed in elderly, males, malnourished, known diabetics, smokers, and alcohol users. (p<0.001). To prevent TB recurrence, all treated tuberculosis patients must be followed at least for 24 months, with screening for Chest X-ray, liquid culture every 6 months, smoking cessation, alcohol cessation, nutritional interventions and good diabetic management.

## Introduction

Recurrent episodes of tuberculosis (RTB) is a public health challenge and has immense implications for TB control worldwide [1]. In areas of high TB and HIV burden, the recurrence is mainly due to exogenous reinfection while in low burden settings, the recurrence is primarily due to endogenous reactivation [2,3]. In India, the proportion of retreatment patients of smear positive pulmonary TB is 24% under the National TB Elimination Programme (NTEP) [4].

Risk of recurrent TB is high among patients who have recently completed treatment for TB. Individuals with recurrent TB are less likely to complete their treatment and also have a higher risk of mortality when compared with those with first episode of TB [5]. Hence recurrent TB patients needs to be identified not only for effective treatment but also to curtail the transmission of disease and prevent mortality. Rate of recurrent TB can be used as a proxy measure to assess the effectiveness of TB control programme in the country and emphasizes the emergence of drug resistance in the community [6]. In addition it increases the ongoing transmission and burden to the health system. Consequently recurrent TB is of high public health importance due to emergence of drug resistance, increased mortality. In this context it is important to understand the burden and epidemiological factors associated with TB recurrence in India, a country with the highest TB burden in the world. This knowledge can be incorporated by the NTEP in its implementation of TB control for achieving END TB goals. We present here the burden of TB recurrence in India based on the world’s largest ever National TB prevalence survey conducted in India, during 2019–2021 [7].

## Methods

### Survey setting, design and procedures

The NTEP in India recommends sputum testing and chest X-ray (CXR) for presumptive TB patients (those with symptoms of cough> 2weeks, fever> 2weeks, significant weight loss and haemoptysis). Those who are sputum smear positive are classified as microbiologically confirmed pulmonary TB (MCPTB). Those who are smear negative but CXR suggestive of TB and those with high clinical suspicion undergo cartridge based nucleic acid amplification test (CBNAAT) and subjected to universal Drug Susceptibility Testing (UDST) in order to start appropriate regimen for the TB patients. The programme also recommends follow up for 24 months after treatment completion.

A nationwide survey to estimate the prevalence of MCPTB among adults>15 years of age was conducted by ICMR-National Institute for Research in Tuberculosis (ICMR-NIRT) along with other ICMR institutes, NTEP, Central TB Division (CTD), National Institute of Tuberculosis and Respiratory Diseases, National Tuberculosis Institute, Intermediate Reference laboratories (IRLs), State TB Cells and WHO- India between June 2019 to September 2021. This was a cross sectional survey using multistage cluster sampling conducted in 443 clusters with a sample size of 800 in each cluster. Eligible participants underwent symptom screening and CXR and those with symptoms suggestive of TB or with history of previous/ current TB treatment and individuals having abnormal CXR underwent testing for TB by sputum CBNAAT, smear microscopy and liquid culture. All the participants were interviewed about their past episode of TB including time and place of treatment and those with reported history of TB treatment in the past were categorized as “Past TB” cases. For the survey “Recurrent TB (RTB)” was defined as participants identified as MCPTB during survey and/or on current TB treatment among those with a reported history of TB treatment in the past and “Non-Recurrent TB (NRTB)” were defined participants with a history of past TB treatment but were not identified as MCPTB and were not on TB treatment during the survey period. All the data were captured electronically. The investigators had access to information that could identify individual participants during and after data collection.

### Data analysis

Descriptive analysis for summarizing the characteristics of survey participants was performed based on "past TB status" and/or "TB history" and RTB. All the statistical analysis were done using Stata16 (Stata Corporation, College Station, TX, USA). The crude prevalence per 100,000 of recurrent TB was estimated along with the confidence interval using the exact binomial formula. Univariate and multiple logistic regression analyses were performed for associations between RTB and risk factors such as age, gender, BMI, smoking, alcohol consumption, TB symptoms, and CXR abnormality. The multivariable analysis included variables known to be associated with PTB identified a-priori by literature review and post-hoc by exploratory data analysis. The statistical difference in duration between the past and current TB diagnosis was compared using an independent t-test. We calculated all estimates and 95% confidence intervals using the Stata *svy* commands to correct for design-effect. All the tests were two-sided, with a type I error set at a = 0.05.

### Ethical statement

The survey was approved by the Institutional Ethics Committee of ICMR- National Institute for Research in Tuberculosis and all other participating institutes. Approval number: 334/ NIRT- IEC/2018 dated 26th November 2018. All Participants above 18 years provided a written informed consent and for participants from 15 years to 18 years written informed assent and Parent’s/ legally authorized representatives’ consent was obtained.

## Results

**Fig 1:**
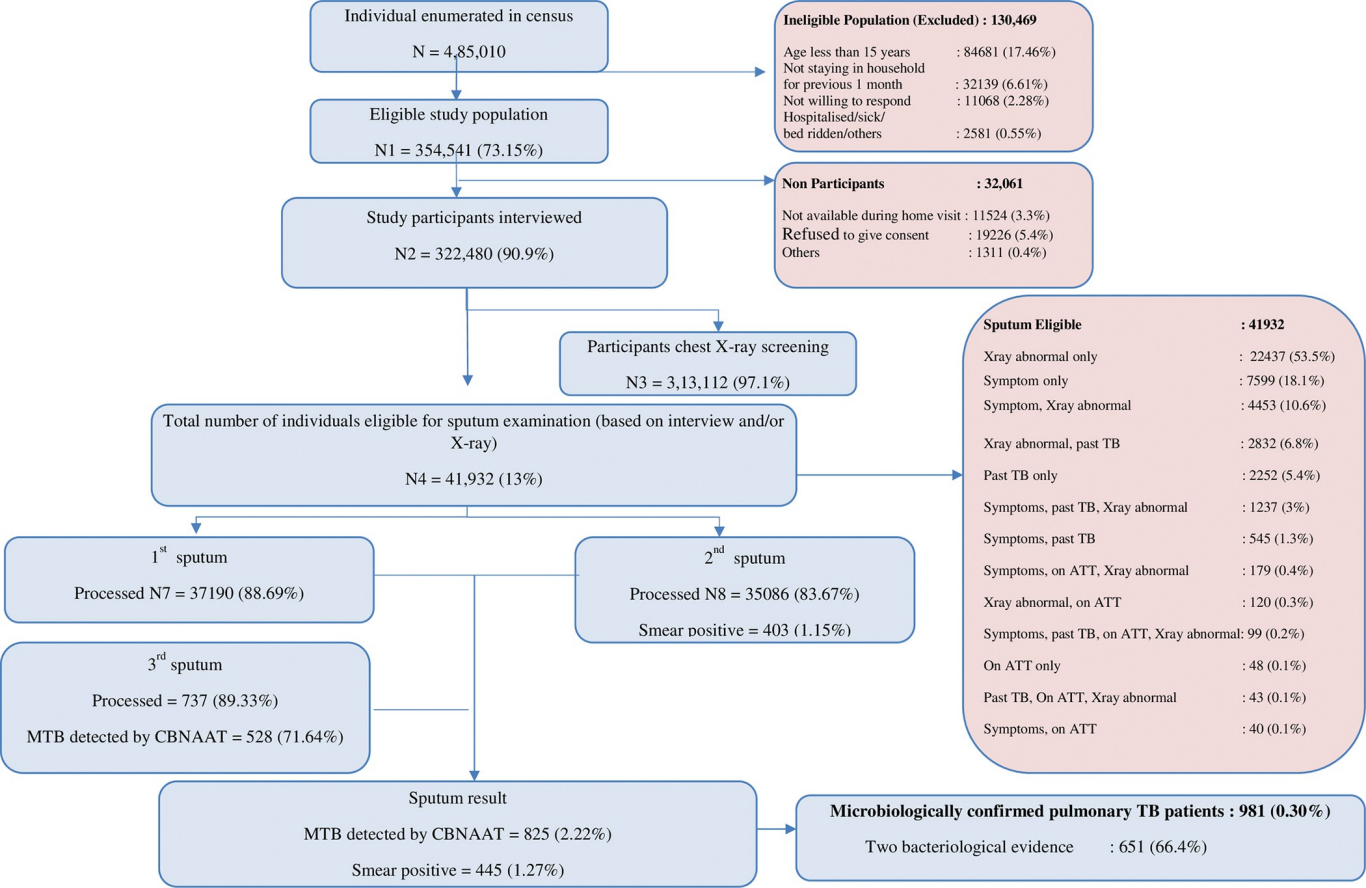
Flow of participants observed in National TB prevalence survey, India, 2019–21.

**Fig 2:**
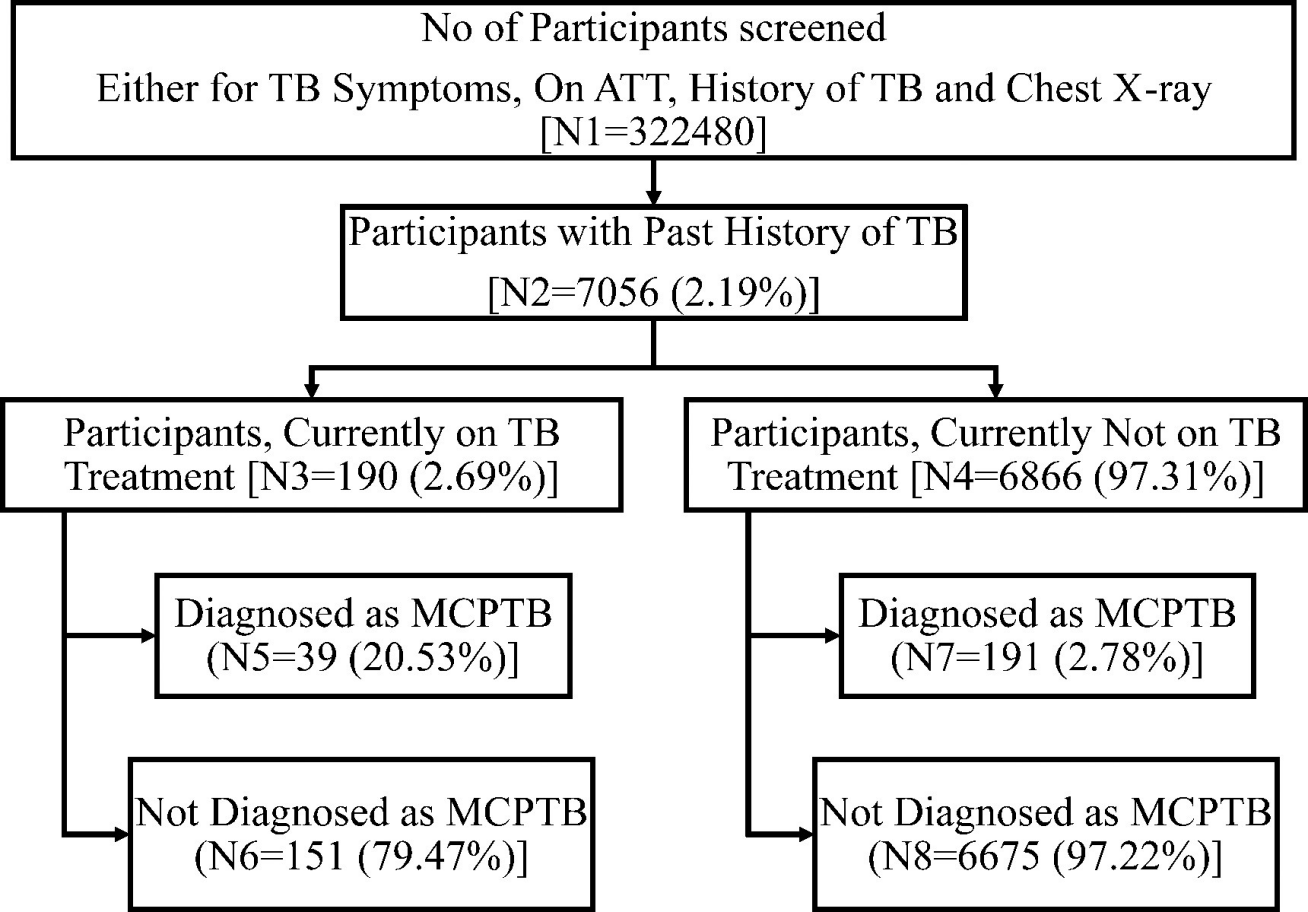
Flowchart showing the recurrent TB cases in the survey.

A total of 354,541 individuals aged more than 15 years participated in the survey and of these 322,480 (90.9%) underwent symptom screening and/or CXR (Fig 1) We identified 7056 (2.2%) participants with a past history of TB, of whom 381 (5.3%) participants were identified as recurrent TB as defined in the survey. Of these 381 patients, 160 (42%) were diagnosed by at least two bacteriological evidence during survey, 70 (18.4%) were diagnosed with one bacteriological and one radiological evidence during survey and 151 (39.6%) were on current TB treatment at the time of survey. Among the 381 recurrent TB cases, 191 (50.1%) were currently not on any treatment and were diagnosed as part of the survey activities. (Fig 2) The crude prevalence (95% CI) of RTB was 118 (107–131) per 100,000 population. The RTB patients (n = 381) contributed to 27.1% of the total (n = 1402) TB patients identified in the survey.

The characteristics of the participants with RTB and its crude prevalence is given in Table 1. The prevalence of RTB was more among the 45 to 64 years age group, males, urban residents, extremely underweight, those with past history of alcohol and smoking and diabetics.

**Table 1:** Characteristics of the participants with recurrent TB and its crude prevalence.

	Overall	Past TB	Recurrent TB	Crudeb Prevalence of Recurrent TB
	n (%a)	n (%a)	n (%a)	per 100,000 (95% CI)
Overall	3,22,480	7056 (2.2)	381 (5.4)	118 (107–131)
**Age**				
15 to 24	66647 (20.7)	505 (7.2)	38 (10)	57 (40–78)
25 to 34	62021 (19.2)	946 (13.4)	58 (15.2)	94 (71–121)
35 to 44	62023 (19.2)	1449 (20.5)	68 (17.8)	110 (85–139)
45 to 54	54590 (16.9)	1602 (22.7)	85 (22.3)	156 (124–192)
55 to 64	43995 (13.6)	1394 (19.8)	79 (20.7)	180 (142–224)
≥65	33204 (10.3)	1160 (16.4)	53 (13.9)	160 (120–209)
**Gender**				
Female	180356 (55.9)	2802 (39.7)	98 (25.7)	54 (44–66)
Male	142106 (44.1)	4254 (60.3)	283 (74.3)	199 (177–224)
Transgender	18 (0.01)	0 (0)	0 (0)	
**Area**				
Urban	89368 (27.7)	2429 (34.4)	280 (73.5)	313 (278–352)
Rural	233112 (72.3)	4627 (65.6)	101 (26.5)	43 (35–53)
**Body Mass Index**				
Extremely Underweight [<16.5]	21516 (6.7)	750 (10.6)	91 (23.9)	423 (341–519)
Underweight [16.5–18.4]	44716 (13.9)	1317 (18.7)	96 (25.2)	215 (174–262)
Normal [18.5–24.9]	172242 (53.4)	3667 (52)	158 (41.5)	92 (78–107)
Pre-obesity [25.0–29.9]	62833 (19.5)	1020 (14.5)	29 (7.6)	46 (31–66)
Obesity—I [30.0–34.9]	17320 (5.4)	237 (3.4)	6 (1.6)	35 (13–75)
Obesity—II [35.0–39.9]	3438 (1.1)	62 (0.9)	1 (0.3)	29 (1–162)
Obesity—III [≥40.0]	415 (0.1)	3 (0)	0 (0)	
**Self-Reported Smoking Status**				
No Smoking history	287398 (89.1)	5491 (77.8)	277 (72.7)	96 (85–108)
Past Smoking history	9932 (3.1)	727 (10.3)	57 (15)	574 (435–743)
Current Smoking history	25150 (7.8)	838 (11.9)	47 (12.3)	187 (137–248)
**Self-Reported Alcohol Status**				
Alcohol users	285675 (88.6)	5487 (77.8)	274 (71.9)	96 (85–108)
Alcohol non users	10266 (3.2)	606 (8.6)	57 (15)	555 (421–719)
Current Alcohol users	26539 (8.2)	963 (13.6)	50 (13.1)	188 (140–248)
**Diabetes Mellitus**				
No	283264 (87.8)	6093 (86.4)	326 (85.6)	115 (103–128)
Not Known	24727 (7.7)	445 (6.3)	23 (6)	93 (59–140)
Yes	14489 (4.5)	518 (7.3)	32 (8.4)	221 (151–312)
**Symptoms Severity**				
Nil	308304 (95.6)	5151 (73)	157 (41.2)	51 (43–60)
Any one symptom	6047 (1.9)	668 (9.5)	42 (11)	695 (501–938)
Having more than one symptoms	8129 (2.5)	1237 (17.5)	182 (47.8)	2239 (1928–2584)
**For Current symptom health care sought?**				
No	9020 (2.8)	695 (9.8)	38 (10)	421 (298–578)
Yes	5156 (1.6)	1210 (17.1)	186 (48.8)	3607 (3115–4153)
**Chest X-ray Findings**				
Normal	281563 (87.3)	2799 (39.7)	58 (15.2)	21 (16–27)
Abnormal	31400 (9.7)	4211 (59.7)	321 (84.3)	1022 (914–1140)
Not Known	9517 (3)	46 (0.7)	2 (0.5)	21 (3–76)

NA—Not Applicable; aColumn percentage; bCrude prevalence per l100,000 with binomial exact confidence level. Recurrent TB is defined as the Currently on TB Treatment and or TB Positives out of the participants with the history of TB.

A total of 7056 participants were classified as “Past TB”. Among them, 381 participants were identified as “RTB” and 6675 as “non- recurrent TB” patients. The median duration of recurrence of TB among the past TB patients was 24 months (IQR = 7,104) whereas the median duration of time from the past episode till the survey time point among the non-recurrent group was 108 months (IQR = 36,240) which was statistically significant. The proportion of past TB patients who sought care from public and private health sector were 78.2% (n = 298) and 21.8% (n = 83) respectively. The proportion of males with a habit of smoking was 9.4%, alcohol consumption was 10.8% and both was 16.5% in the recurrent TB group compared to 7.1%, 7.4% and 13.7% respectively in the non-recurrent TB group. This difference was statistically significant both by univariate and multivariate logistic regression. The proportion of RTB patients with diabetes mellitus was 8.4% compared to 7.3% in the non-recurrent group [Table 2]. Out of the 381 RTB patients, 18 (4.7%) had a household contact (HHC) who were diagnosed with TB during the survey.

**Table 2:** Characteristics of the participants with the recurrent TB and Non-recurrent TB.

	Non-Recurrent (n = 6675)	Recurrent (n = 381)	Odds Ratio	pValue	Adj. OR	p value
	n (%^a^)	n (%^a^)	(95% CI)		(95% CI)	
**Age Classification**						
≥60	1438 (21.5)	65 (17.1)	Reference		Reference	
45 to 59	2147 (32.2)	129 (33.9)	1.33 (0.98–1.80)	0.068	1.70 (1.24–2.33)	***0*.*001***
30 to 44	2053 (30.8)	107 (28. 0)	1.15 (0.84–1.58)	0.376	1.90 (1.36–2.66)	**<0.001**
15 to 29	1037 (15.5)	80 (21.0)	1.71 (1.22–2.39)	***0*.*002***	2.89 (2.01–4.16)	**<0.001**
**Gender in combination with Smoking and or Alcohol Usage**						
Female, without Smoke and Alcohol	2601 (39.0)	93 (24.4)	Reference		Reference	
Male, without Smoke and Alcohol	2089 (31.3)	143 (37.5)	1.91 (1.47–2.50)	**<0.001**	1.75 (1.33–2.31)	**<0.001**
Female, Smoking and or Alcohol usage	103 (1.5)	5 (1.3)	1.36 (0.54–3.41)	0.515	1.01 (0.39–2.59)	0.984
Male, Smoke alone	473 (7.1)	36 (9.4)	2.13 (1.43–3.17)	**<0.001**	1.82 (1.20–2.77)	***0*.*005***
Male, Alcohol alone	496 (7.4)	41 (10.8)	2.31 (1.58–3.38)	**<0.001**	2.09 (1.41–3.10)	**<0.001**
Male, Smoke and Alcohol	913 (13.7)	63 (16.6)	1.93 (1.39–2.68)	**<0.001**	1.47 (1.04–2.08)	***0*.*030***
**Diabetes Mellitus**						
No	6189 (92.7)	349 (91.6)	Reference		Reference	
Yes	486 (7.3)	32 (8.4)	1.17 (0.80–1.70)	0.416	1.65 (1.11–2.47)	***0*.*014***
**TB Symptoms and or Body Mass Index**						
No Symptoms and BMI≥18.5	3749 (56.1)	99 (26.0)	Reference		Reference	
Symptoms and BMI≥18.5	1046 (15.7)	95 (24.9)	3.44 (2.57–4.60)	**<0.001**	3.14 (2.34–4.23)	**<0.001**
No Symptoms and BMI<18.5	1245 (18.7)	58 (15.2)	1.76 (1.27–2.46)	**<0.001**	1.58 (1.13–2.21)	***0*.*008***
Symptoms and BMI<18.5	635 (9.5)	129 (33.9)	7.69 (5.84–10.13)	**<0.001**	6.59 (4.95–8.77)	**<0.001**
**Chest X-ray Finding**						
Normal	2785 (41.7)	60 (15.7)	Reference		Reference	
Abnormal	3890 (58.3)	321 (84.3)	3.83 (2.9–5.07)	**<0.001**	3.28 (2.46–4.38)	**<0.001**

NA—Not Applicable; ^a^Column percentage; Adj. OR—Adjusted Odds Ratio.

The survey identified 1021 new patients with no reported past episode of TB. The difference in characteristics between new TB patients and RTB patients is shown in Table 2. The Odds Ratio (95% CI) for RTB among males who smoke was 1.269 (0.812–1.984), males with alcohol consumption was 2.157 (1.37–3.396) and males with both smoking and alcohol usage was 1.399 (0.963–2.032). The proportion of RTB patients with symptoms and BMI<18.5 was 33.9%, symptoms and BMI >18.5 was 24.9%, no symptoms and BMI<18.5 was 15.2% and no symptoms and BMI>18.5 was 26% compared to 23.7%, 13.8%, 18.3% and 23.5% respectively among the newly diagnosed patients. There was a statistically significant (p<0.001) difference in age, gender, self-reported alcohol status, symptoms and BMI between the new and recurrent patients (Table 3). The proportion of drug resistance, identified by Gene-Xpert was more among recurrent MCPTB patients (11.3%) when compared with new MCPTB (4.3%) patients identified in the survey. This difference was statistically significant (p<0.001) with an OR (95% CI) of 3.058 (1.666–5.611).

**Table 3:** Characteristics of the participants with recurrent TB and newly diagnosed TB.

	New Diagnosed TB (n = 1021)	TB Recurrent (n = 381)	Odds Ratio	pValue	Adj. OR	pValue
	n (%^a^)	n (%^a^)	(95% CI)		(95% CI)	
**Age Classification**						
≥60	268 (26.2)	65 (17.0)	Reference		Reference	
45 to 59	295 (28.9)	129 (33.9)	1.80 (1.28–2.54)	<0.001	1.79 (1.27–2.54)	<0.001
30 to 44	200 (19.6)	107 (28.1)	2.21 (1.54–3.16)	<0.001	2.02 (1.40–2.92)	<0.001
15 to 29	258 (25.3)	80 (21.0)	1.28 (0.88–1.85)	0.192	1.32 (0.90–1.95)	0.159
**Gender in combination with Smoking and or Alcohol Usage**						
Female, without Smoke and Alcohol	318 (31.1)	93 (24.4)	Reference		Reference	
Male, without Smoke and Alcohol	366 (35.8)	143 (37.5)	1.34 (0.99–1.81)	0.059	1.34 (0.99–1.83)	0.063
Female, Smoking and or Alcohol usage	21 (2.1)	5 (1.3)	0.81 (0.30–2.22)	0.688	0.81 (0.29–2.24)	0.681
Male, Smoke alone	97 (9.5)	36 (9.4)	1.27 (0.81–1.98)	0.296	1.29 (0.81–2.06)	0.290
Male, Alcohol alone	65 (6.4)	41 (10.8)	2.16 (1.37–3.40)	<0.001	2.11 (1.32–3.37)	0.002
Male, Smoke and Alcohol	154 (15.1)	63 (16.6)	1.40 (0.96–2.03)	0.078	1.29 (0.87–1.90)	0.210
**Diabetes Mellitus**						
No	952 (93.2)	349 (91.6)	Reference		Reference	
Yes	69 (6.8)	32 (8.4)	1.27 (0.82–1.96)	0.291	1.20 (0.76–1.90)	0.429
**TB Symptoms and or Body Mass Index**						
No Symptoms and BMI≥18.5	330 (32.3)	99 (26.0)	Reference		Reference	
Symptoms and BMI≥18.5	193 (18.9)	95 (24.9)	1.64 (1.18–2.29)	0.004	1.54 (1.10–2.17)	0.013
No Symptoms and BMI<18.5	256 (25.1)	58 (15.2)	0.76 (0.53–1.09)	0.129	0.79 (0.55–1.15)	0.218
Symptoms and BMI<18.5	242 (23.7)	129 (33.9)	1.78 (1.30–2.42)	<0.001	1.80 (1.31–2.47)	<0.001
**Chest X-ray Finding**						
Normal	105 (10.3)	60 (15.7)	Reference		Reference	
Abnormal	916 (89.7)	321 (84.3)	0.61 (0.44–0.86)	0.005	0.63 (0.44–0.90)	0.011

^a^Column percentage; Adj. OR—Adjusted Odds Ratio.

## Discussion

The RTB observed in the current survey is high, in terms of absolute numbers, for a country like India. The proportion of RTB patients identified in the survey is slightly higher than proportion of sputum smear positive retreatment patients (24%) as reported in the NTEP [8] This is higher than the reported proportion of relapse in Zambia and other African counties [9]. of TB indicate that patients were cured but the underlying medical or social conditions have not been addressed completely. Factors like malnutrition, diabetic control, alcohol consumption and smoking that probably led to the previous episode of TB have not been addressed completely which is very essential to be tackled as we march towards TB elimination [10] RTB cases contributes to more than one fourth of the burden of TB prevalence in India. This emphasis the need for devising further strategies to closely monitor TB patients who have completed treatment. This will help early diagnosis of TB recurrence to prevent emergence of drug resistance and mortality. The prevalence of RTB is more in males (approximately 4 times) than in females. Similar findings were observed in the overall prevalence in the survey as well as in other studies [11,12]. This was in contrary to a systematic review done earlier in India which showed that there was no gender difference in recurrence [5].

Residing in an urban area, those with a reported past or current smoking status, past or current alcohol user, presence of DM and BMI<18 were all risk factors for RTB. All these are known risk factors for PTB and it would be reasonable to say that the trend applies here too. Similar findings have been reported in various other studies too [11–14]. The median duration of past TB episode from the survey time point in those with recurrent TB was 24 months which aligns with NTEP’s policy to follow TB patients for 2-years after treatment completion. A seven-years prospective study done in China showed that 76% of recurrence occurs in the first 3 years [12]. Similar results were noted in few other studies [15,16]. We also found that around 11% of recurrent patients had only one symptom. This emphasizes that people who had an episode of TB should be carefully followed up for presumptive TB symptoms and even a single symptom should not be ignored to identify recurrence at the earliest. It should be taken into consideration that past TB patients might have old lesions which would have been reported as X-ray abnormality in the survey. Inclusion of X-ray should be considered in the post treatment follow up strategy since nearly 41.2% of recurrent TB patients did not report any symptoms and identification of any new lesion can pick up the recurrence at the early stage of the disease. Though the participants had a past history of TB, we found that 17% of those with symptoms did not seek health care. The various delays in health seeking behaviour of TB patients may lead to disastrous outcomes like, drug resistance TB and this is even more likely in patients with recurrent TB [17]. There is a high proportion of MDR TB patients among the recurrent TB patients stressing the need for focused interventions to prevent TB recurrence and circumvent drug resistant TB. The prevalence of MDR-TB among previously treated TB cases in the survey (11.3%) is similar to the findings from another Indian study where the estimated prevalence is 12 to 14% [18]. From a public health perspective, recurrent TB patients contributes to the ongoing TB transmission to their contacts at home and community. Moreover, the higher risk of drug resistant TB makes it even more important to identify them at the earliest to combat the growing burden of drug resistant TB in the country [19].

The study has identified many risk factors for recurrence of TB, which can help in targeted post tuberculosis care and monitoring. Various studies conclude that active smoking increase the risk of TB relapse and also states that smokers were less likely to adhere to the tuberculosis treatment [20]. Around half of the patients with past history of TB (191/381) would have missed TB diagnosis at the National Level in the absence of this survey, indicates gaps in case detection and follow-up among the TB treated patients. When translated to absolute numbers, this may contribute to significant amount of TB transmission, drug resistance and mortality. Similar situation may be found in other high TB burden countries. In the context of moving towards END TB Goals, NTPs in India and other high burden countries, should strengthen the implementation of Post treatment follow-up among TB treated patients, for early diagnosis of recurrent TB, prevention of TB transmission and TB mortality.

## Limitations

The recurrent TB was based on self-reporting is a huge limitation, there would be others who had recurrent TB but either did not report or died before diagnosis. The actual proportion of TB patients who experience recurrence may be different in fact higher than 5.3%, the observed figure in this study. The study could not distinguish as to whether these recurrences were due to relapse or reinfection. We did not capture details about the past TB illness in terms of type of TB disease, treatment details, smear status, outcome of the treatment which would have provided much more insights about the factors for TB recurrence. On the other hand, the operational definition used in the survey for MCPTB cases definition may overestimate the recurrent patients to a small extent because CBNAAT and smear may report positive for dead bacilli too. We expect that due to the composite nature of the case definition of MCPTB this would have been minimized.

## Conclusion

Given the burden of increasing drug resistance in India, there is need for specific strategies to address risk factors for TB recurrence. The implementation gaps in post treatment follow up among TB treated patients for at least 24 months should be addressed. Health education awareness about benefits of smoking and alcohol cessation to TB patient must be offered to all TB patients. Emphasis on nutritional interventions and good diabetic control in the post treatment follow-up to prevent TB recurrence should be considered.

X-ray should be included in the follow up protocols to identify any new lesions. There is a growing need for effective vaccines or immunomodulators for post treatment prophylaxis to prevent recurrence by boosting immunity. Given the high cost of treatment that programme incurs for each patient in India, it is crucial to control recurrence of TB in treated patients to prevent drug resistance and TB transmission from these patients.
